# Archaerhodopsin Selectively and Reversibly Silences Synaptic Transmission through Altered pH

**DOI:** 10.1016/j.celrep.2016.07.057

**Published:** 2016-08-11

**Authors:** Mohamady El-Gaby, Yu Zhang, Konstantin Wolf, Christof J. Schwiening, Ole Paulsen, Olivia A. Shipton

**Affiliations:** 1 Department of Physiology, Development and Neuroscience, University of Cambridge, Cambridge CB2 3EG, UK

**Keywords:** optogenetics, archaerhodopsin, pH, synaptic silencing, synapses, reversible, behavior, long-term memory, hippocampus, asymmetry

## Abstract

Tools that allow acute and selective silencing of synaptic transmission in vivo would be invaluable for understanding the synaptic basis of specific behaviors. Here, we show that presynaptic expression of the proton pump archaerhodopsin enables robust, selective, and reversible optogenetic synaptic silencing with rapid onset and offset. Two-photon fluorescence imaging revealed that this effect is accompanied by a transient increase in pH restricted to archaerhodopsin-expressing boutons. Crucially, clamping intracellular pH abolished synaptic silencing without affecting the archaerhodopsin-mediated hyperpolarizing current, indicating that changes in pH mediate the synaptic silencing effect. To verify the utility of this technique, we used trial-limited, archaerhodopsin-mediated silencing to uncover a requirement for CA3-CA1 synapses whose afferents originate from the left CA3, but not those from the right CA3, for performance on a long-term memory task. These results highlight optogenetic, pH-mediated silencing of synaptic transmission as a spatiotemporally selective approach to dissecting synaptic function in behaving animals.

## Highlights

• Archaerhodopsin selectively and reversibly silences synaptic transmission
• Archaerhodopsin silences synaptic transmission without blocking action potentials
• Archaerhodopsin mediates synaptic silencing through changes in pH
• Synaptic silencing reveals distinctions among CA3-CA1 synapses during learning

## Graphical Abstract

**Figure:**
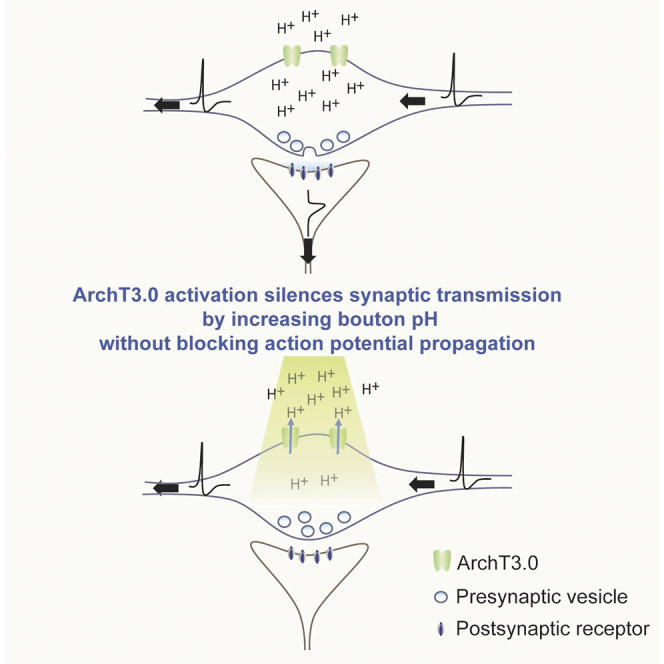

## Teaser

El-Gaby et al. demonstrate that archaerhodopsin can acutely and selectively silence synaptic transmission through changes in pH rather than hyperpolarization. Application of this tool in behaving animals reveals a necessity for synapses from the left CA3 onto CA1 neurons, but not from the right CA3, in long-term memory performance.

## Introduction

Optogenetic tools have ushered in a new era in the quest to understand the neural basis of behavior. Through rapid, reversible, and cell-type-specific silencing of neuronal firing, light-driven hyperpolarizing ion pumps have enabled a hitherto unparalleled spatiotemporal precision in interrogating neuronal populations and their involvement in specific behaviors (Yizhar et al., 2011, Goshen et al., 2011, Shipton et al., 2014). Similarly, methods to silence synaptic transmission with high spatiotemporal precision would be of great value in assessing the roles of specific synaptic connections in behavior (e.g., Martin et al., 2000, Rolls, 2010, Larkum, 2013). Efforts to address this tool gap include the recent development of an optogenetic synaptic silencing method, termed chromophore assisted light inactivation (CALI; Lin et al., 2013). CALI acts via oxidative disruption of synaptic release machinery and has enabled light-mediated reduction of synaptic transmission in *Caenorhabditis elegans* and concomitant disruption of movement (Lin et al., 2013). However, the recovery of synaptic transmission after such a manipulation is incomplete and slow, requiring at least 24 hr for movement to re-emerge (Lin et al., 2013), precluding repeated use for multiple behavioral trials. Furthermore, manipulations with such prolonged effects may trigger compensatory changes at the subcellular, cellular, and network levels (Turrigiano, 2008, Goshen et al., 2011), which could confound the interpretation of synaptic silencing experiments. Thus, in order to fully harness the power of optogenetics for understanding the behavioral function of specific synapses, there is a need for rapidly reversible synaptic silencing tools.

A potentially promising approach for acute synaptic silencing makes use of the halorubrum-derived opsins, archaerhodopsin (Arch) and archaerhodopsin T (ArchT). These opsins respond to yellow or green light by pumping protons out of cells, thereby producing membrane hyperpolarization (Chow et al., 2010, Han et al., 2011). Compared to equivalent versions of other commonly used silencing opsins, archaerhodopsins have higher maximum photocurrents, increased light sensitivity, and strong expression in the axonal plasma membrane (Chow et al., 2010, Han et al., 2011), which are further enhanced for third-generation versions (Arch3.0 and ArchT3.0) by the addition of endoplasmic reticulum export motifs and neurite targeting sequences (Mattis et al., 2011). These factors make archaerhodopsins potentially suitable for acute silencing of axonal outputs. Furthermore, this approach builds on the successful use of optogenetics in mammalian systems, where the prevalence and complexity of compensatory mechanisms places a premium on manipulations with rapid onset and offset (Goshen et al., 2011). We therefore investigated the effects of axonal archaerhodopsin activation on synaptic transmission and the mechanisms through which archaerhodopsins may act at axonal projections. We demonstrate that archaerhodopsins are capable of robust, rapid, and reversible synaptic silencing. Surprisingly, this effect was mediated via changes in pH rather than hyperpolarization. Furthermore, we employ trial-limited, archaerhodopsin-mediated presynaptic silencing in vivo to demonstrate a differential requirement of distinct subpopulations of CA3-CA1 synapses in hippocampus-dependent learning.

## Results

### Archaerhodopsin Produces Robust Reversible Silencing of Synaptic Transmission

**Figure 1:**
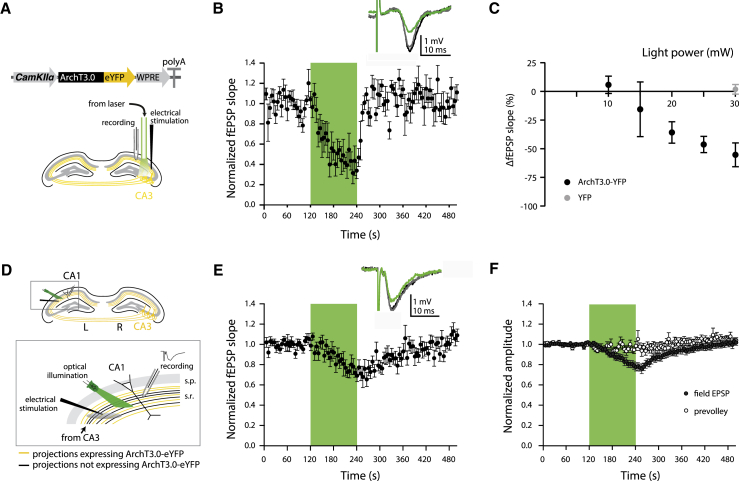
Optogenetic Synaptic Silencing In Vivo and Ex Vivo (A) Top: adeno-associated virus containing an ArchT3.0-eYFP construct under the control of a CaMKIIα promoter was unilaterally injected into the dorsal CA3 area of C57BL/6J mice. Bottom: electrical stimulation was delivered in the stratum radiatum of the CA1 of urethane-anaesthetized mice contralateral to the injected hemisphere. An optical fiber attached to a 532-nm (green) laser illuminated electrically stimulated axons. An ACSF-filled glass electrode was used to record fEPSPs in vivo. (B) Green light (532 nm, 30 mW) delivered for 2 min caused a reversible reduction in the normalized fEPSP slope. Green area represents time of light delivery. Inset: representative fEPSPs before (black), during (green), and after (gray) light delivery. (C) Light-induced change in fEPSP slope in ArchT3.0-eYFP-expressing mice is light intensity dependent. Green light delivery (30 mW) in mice expressing eYFP only did not cause any changes in the fEPSP slope. (D) Electrical stimulation was delivered in the stratum radiatum of CA1 in coronal slices from mice with unilateral CA3 expression of ArchT3.0-eYFP. An optical fiber attached to a 532-nm laser illuminated electrically stimulated axons. An ACSF-filled glass electrode was used to record in vitro fEPSPs. (E) Green light (532 nm, 2 mW) delivered for 2 min caused a reversible reduction in normalized fEPSP slope. Inset: representative fEPSPs before (black), during (green), and after (gray) light delivery. (F) Light delivery causes a reversible reduction in normalized fEPSP amplitude without affecting the amplitude of the presynaptic fiber volley. Error bars represent mean ± SEM. See also Figure S1.

To assess the ability of light-driven proton pumps to achieve silencing of synaptic transmission, we stereotactically injected an adeno-associated viral construct encoding ArchT3.0-eYFP under the control of the calcium/calmodulin-dependent protein kinase IIα (CaMKIIα) promoter (Mattis et al., 2011) unilaterally into mouse CA3 (Figure 1A). An eYFP-only construct was used for control mice. We subsequently recorded field excitatory postsynaptic potentials (fEPSPs) from Schaffer collateral stimulation in the CA1 stratum radiatum (containing ArchT3.0-eYFP-expressing CA3 axons) of urethane anaesthetized animals and delivered green (532-nm) light to CA3-CA1 axonal projections (Figure 1A). Light delivery in mice expressing ArchT3.0-eYFP produced robust, reversible reduction of fEPSPs (light-induced change in fEPSP slope [ΔfEPSP slope] at 30-mW, 532-nm light: −55.3% ± 10.4%; n = 5; one-way ANOVA: F = 29.6, p < 0.001; Tukey post hoc test: light off(pre) versus light on p < 0.001, light off(post) versus light on p < 0.001, light off(pre) versus light off(post) p = 0.619; Figure 1B). In contrast, this effect was not present in eYFP-only control mice (ΔfEPSP slope at 30-mW, 532-nm light: +1.9% ± 4.2%; n = 4; one-way ANOVA: F = 0.062, p = 0.940; unpaired two-tailed t test, ArchT3.0-eYFP versus control eYFP-only: p = 0.002; Figures 1B, 1C, and S1A). The ArchT3.0-mediated reduction and recovery of fEPSPs occurred on a timescale of seconds (at 30-mW light intensity: reduction time constant, 18.4 ± 5.9 s; recovery time constant, 13.1 ± 1.3 s; n = 5; Figure 1B). Furthermore, the magnitude of this effect varied with light intensity (ΔfEPSP slope: +5.8% ± 7.6% at 10 mW, −15.6% ± 23.8% at 15 mW, −35.8% ± 9.4% at 20 mW, −46.3% ± 7.1% at 25 mW, −55.3% ± 10.4% at 30 mW; n = 3–5; one-way ANOVA: F = 4.80, p = 0.011; Figure 1C).

To enable mechanistic studies, we turned to the hippocampal slice preparation. We recorded from the CA1 stratum radiatum in coronal hippocampal slices from mice expressing ArchT3.0-eYFP (Figure 1D) and observed a qualitatively similar fEPSP reduction to that observed in vivo (ΔfEPSP slope at 2-mW, 532-nm light: −21.3% ± 6.6%; n = 19; one-way ANOVA: F = 7.42, p = 0.001; Tukey post hoc test: light off(pre) versus light on p = 0.002, light off(post) versus light on p = 0.008, light off(pre) versus light off(post) p = 0.911; Figure 1E). Again, no effect was observed in control mice expressing eYFP alone (ΔfEPSP slope at 2-mW, 532-nm light: +2.0% ± 3.5%; n = 7; one-way ANOVA: F = 0.806, p = 0.462; unpaired two-tailed t test, ArchT3.0-eYFP versus control eYFP-only: p = 0.048; Figure S1B). The fEPSP amplitude showed a similarly reversible ArchT3.0-mediated reduction (ΔfEPSP amplitude at 2 mW: −16.7% ± 3.5%; n = 19; one-way ANOVA: F = 12.8, p < 0.001; Tukey post hoc test: light off(pre) versus light on p < 0.001, light off(post) versus light on p < 0.001, light off(pre) versus light off(post) p = 0.970; Figure 1F). Importantly, despite the clear effect on fEPSP slope and amplitude, the amplitude of the extracellularly recorded, evoked presynaptic fiber volley was unaffected by ArchT3.0 activation, indicating that the silencing was restricted to synaptic transmission (Δpresynaptic volley amplitude: −2.6% ± 3.1%; n = 19; one-way ANOVA: F = 0.718, p = 0.492; Figure 1F).

### Silencing of Synaptic Transmission Is Accompanied by an Increase in pH inside Archaerhodopsin-Expressing Presynaptic Boutons

**Figure 2:**
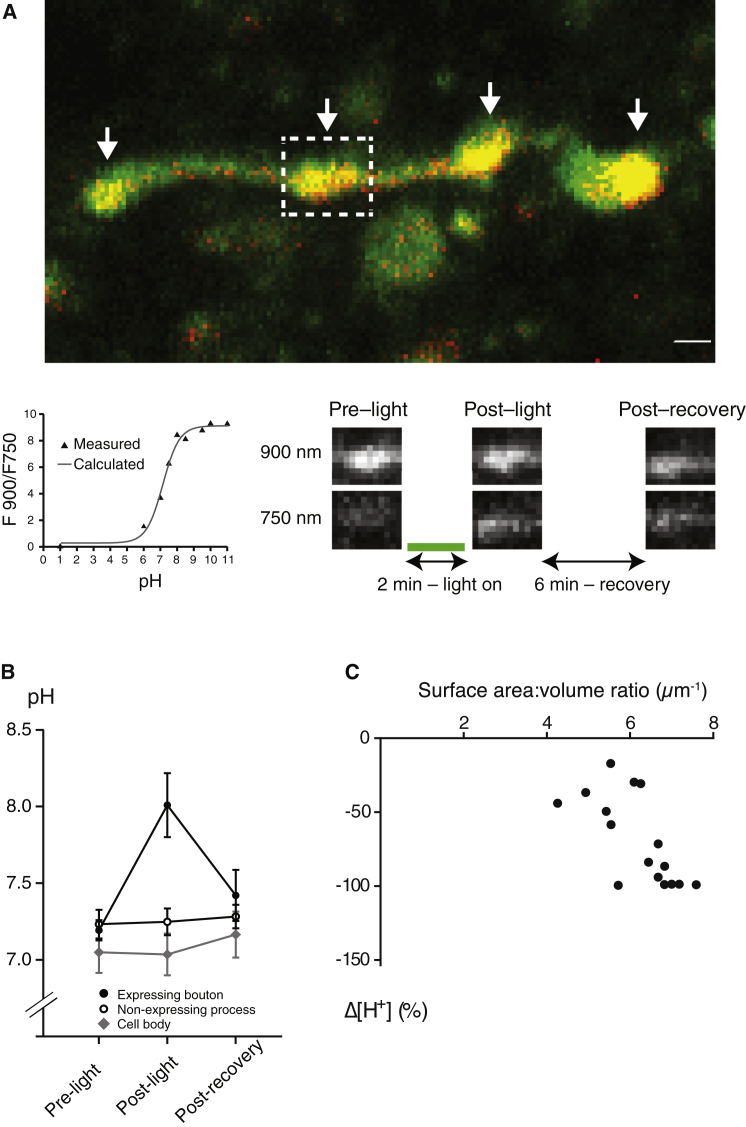
ArchT3.0 Activation in CA3-CA1 Axons Results in pH Transients that Are Restricted to Expressing Boutons (A) Two-photon bouton pH imaging procedure. Upper: ArchT3.0-eYFP-expressing boutons (white arrows) were identified as boutons showing fluorescence at both 800 nm (green; 8-hydroxypyrene-1,3,6-trisulfonic acid [HPTS)] and 980 nm (red; eYFP). Lower left: calibration curve showing the measured HPTS fluorescence ratio (with a 525-nm filter) when exciting at 900 and 750 nm (two-photon) plotted at known pH values and fit with a sigmoidal function. Lower right: green (532-nm single-photon) light was delivered at 2 mW for 2 min. Fluorescence was measured with a 525-nm filter at 900 and 750 nm (two-photon) excitation immediately before light delivery (pre-light), immediately after light cessation (post-light), and 6 min after light cessation (post-recovery). The 900/750 fluorescence ratio was then used to calculate intra-bouton pH in each condition. (B) Pre-light, post-light, and post-recovery pH measurements in ArchT3.0-eYFP-expressing CA3-CA1 boutons, neighboring non-expressing processes in the CA1 stratum radiatum, and ArchT3.0-eYFP-expressing CA3 somata. (C) Plot showing correlation between estimated bouton surface-area-to-volume ratio and light-induced percentage change in bouton [H^+^] for ArchT3.0-eYFP-expressing boutons. Error bars represent mean ± SEM.

The time course of silencing of synaptic transmission and the lack of an effect on the presynaptic action potential both suggest that hyperpolarization does not play a major role in the action of ArchT3.0 at axonal projections. Therefore, we considered the possibility that pH changes in presynaptic boutons might be the mechanism for ArchT3.0-mediated synaptic silencing. To investigate this, we incorporated the pH indicator 8-hydroxypyrene-1,3,6-trisulfonic acid (HPTS) into slices from mice expressing ArchT3.0-eYFP in CA3 neurons. The pH was estimated from the ratio of fluorescence with two-photon excitation at 900 nm and 750 nm (900/750 ratio; Figure 2A; see Experimental Procedures). In support of previous suggestions that there are limited pH changes at the neuronal cell body in response to archaerhodopsin activation (Chow et al., 2010), we found no significant pH change when ArchT3.0 was activated at the soma of CA3 neurons by (single-photon) delivery of 532-nm light for 2 min (ΔpH = −0.01 ± 0.07; n = 7; one-way ANOVA: F = 0.197, p = 0.823; Figure 2B). However, when we restricted light delivery to axonal projections from the CA3 in the CA1, we found a robust and reversible increase in pH at boutons expressing ArchT3.0-eYFP (ΔpH = +0.82 ± 0.20; n = 18; one-way ANOVA: F = 7.03, p = 0.002; Tukey post hoc test: light off(pre) versus light on p = 0.002, light off(post) versus light on p = 0.030, light off(pre) versus light off(post) p = 0.574; unpaired two-tailed t test, soma versus boutons: p = 0.015; Figure 2B). This effect was highly selective, since neighboring processes that were similarly sized but did not express ArchT3.0-eYFP exhibited no change in pH upon light delivery (ΔpH = +0.02 ± 0.07; n = 15; one-way ANOVA: F = 0.088, p = 0.916; unpaired two-tailed t test, ArchT3.0-eYFP-expressing versus non-expressing processes: p = 0.001; Figure 2B). Equivalent results were seen after correcting for the possible contribution of eYFP fluorescence to ΔpH values (see Experimental Procedures). Furthermore, there was no significant change in the 900/750 fluorescence ratio of boutons expressing eYFP only that had taken up HPTS when light was delivered (light off (pre): 11.1 ± 1.1; light on: 10.0 ± 1.2; light off (post): 10.7 ± 1.4; one-way ANOVA: F = 0.547, p = 0.584), in contrast to ArchT3.0-eYFP-expressing boutons (900/750 ratio, ArchT-expressing boutons: light off (pre) 4.87 ± 0.26, light on 6.88 ± 0.42, light off (post) 5.36 ± 0.40; one-way ANOVA: F = 8.14, p = 0.001; Tukey post hoc test: light off(pre)-light on p = 0.001, light off(post)-light on p = 0.014, light off(pre)-light off(post) p = 0.612), confirming that ArchT3.0 expression is necessary for the light-induced increase in bouton pH.

We hypothesized that a pH change was observed at boutons, but not the soma, due to the higher plasma membrane surface-area-to-volume (SAV) ratio in boutons and hence potentially a higher ArchT3.0-mediated outward proton efflux per unit volume. Consistent with this hypothesis, we observed a significant correlation between bouton SAV ratio (estimated from cross-sectional area; see Experimental Procedures) and light-induced % change in [H^+^] (Spearman’s rank correlation: r = 0.551, p = 0.018; Figure 2C). Furthermore, by factoring in the measured pH change at boutons and estimated SAV ratios for both reconstructed boutons and the smallest soma imaged, and assuming similar plasma-membrane ArchT3.0 density and buffering capacities for both compartments, the theoretically expected pH change at the soma under our illumination conditions was 0.02 pH units (see Experimental Procedures).

### Changes in pH Are Necessary for Archaerhodopsin-Mediated Silencing of Synaptic Transmission

**Figure 3:**
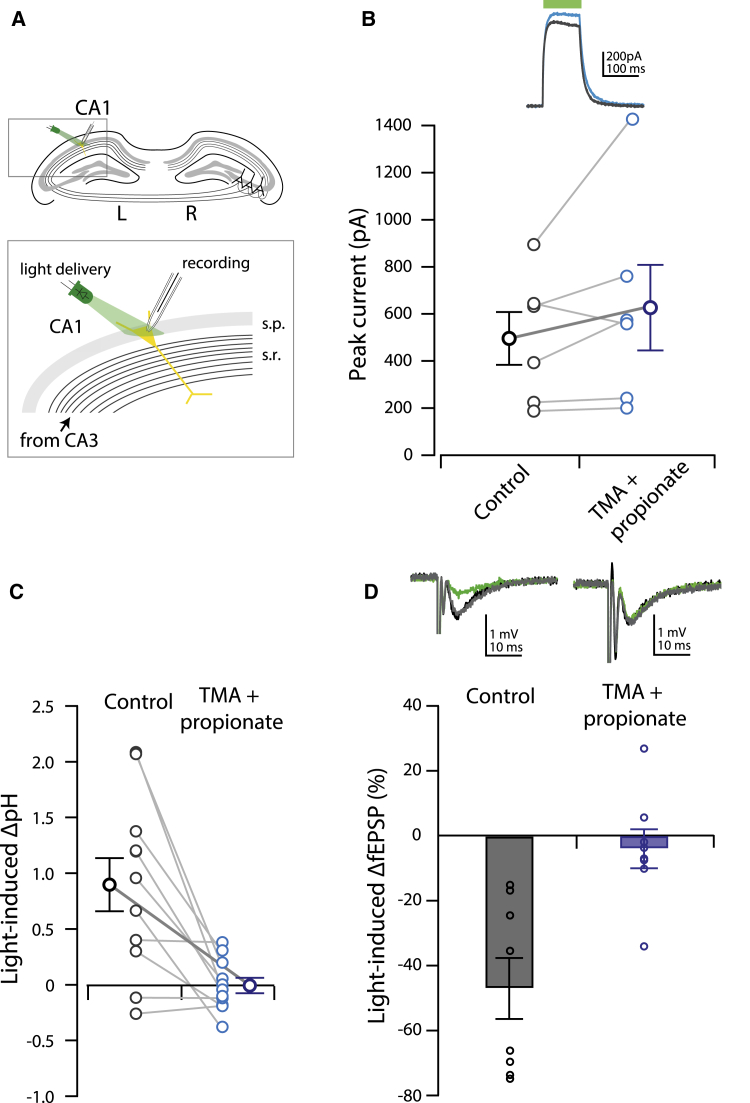
Clamping Intracellular pH Blocks Optogenetic Synaptic Silencing (A) Experimental setup for ArchT3.0 current measurement: ArchT3.0-expressing CA1 pyramidal neurons were patched and an optical fiber attached to a 532-nm laser was used to deliver light close to the soma. 100-ms pulses of 2-mW light were delivered and resultant currents measured in voltage-clamp mode. (B) Peak ArchT3.0 current was not affected by isosmotic addition of weak acid (propionate) and weak base (trimethylamine; TMA) to HEPES-based control ACSF. Inset: representative ArchT3.0 current traces in HEPES control (black) and TMA + propionate (blue). (C) Light-induced change in ArchT-eYFP-expressing CA3 bouton pH is abolished by isosmotic addition of propionate and TMA to HEPES-based ACSF. (D) Light-induced change in fEPSP slope in ArchT-eYFP-expressing CA3-CA1 axons is abolished by isosmotic addition of propionate and TMA to HEPES-based ACSF. Inset: representative fEPSPs before (black), during (green), and after (gray) light delivery in HEPES control (left) and TMA + propionate (right) solutions. Error bars represent mean ± SEM.

To investigate the mechanism through which ArchT3.0 acts at synapses, we sought to selectively block the ArchT3.0-mediated increase in bouton pH without reducing its hyperpolarizing current. Compared to a HEPES-based control solution, isosmotic addition of the membrane permeable acid and base, propionic acid (20 mM) and trimethyl amine (TMA; 3 mM; Pantazis et al., 2006), had no effect on the hyperpolarizing current mediated by ArchT3.0 at the soma (ArchT3.0-mediated current: HEPES control = 457 ± 112 pA, HEPES + TMA + propionate = 587 ± 181 pA; n = 6; paired two-tailed t test, HEPES control versus pH clamped: p = 0.200; Figures 3A and 3B). However, while a robust pH change was observed in ArchT3.0-eYFP-expressing boutons following ArchT3.0-activation in the control HEPES-based solution, isosmotic addition of propionate and TMA (as above) blocked this pH change within the same boutons (ΔpH HEPES control = 0.90 ± 0.24; ΔpH pH clamped = −0.01 ± 0.07; n = 11; paired two-tailed t test, HEPES control versus pH clamped: p = 0.002; Figure 3C), without significantly affecting their baseline pH (HEPES control pH = 7.27 ± 0.16; pH clamped = 7.11 ± 0.06; paired two-tailed t test, HEPES control versus pH clamped: p = 0.441). A similar result was seen when correcting for the possible contribution of eYFP fluorescence to ΔpH values (see Experimental Procedures). Thus, the propionate and TMA solution used here selectively blocks ArchT3.0-mediated bouton alkalinization, effectively “clamping” internal pH, without affecting the ArchT3.0-mediated hyperpolarizing current.

We next used pH clamping to investigate whether reduction of fEPSPs by ArchT3.0 was mediated by changes in pH. ArchT3.0-mediated synaptic silencing was robust in a control HEPES-based solution (ΔfEPSP slope in HEPES control: −47.0% ± 9.4%; n = 8; one-way ANOVA: F = 15.1, p < 0.001; Tukey post hoc test: light off(pre) versus light on p < 0.001, light off(post) versus light on p < 0.001, light off(pre) versus light off(post) p = 0.796; Figure 3D). However, clamping intracellular pH was sufficient to abolish ArchT3.0-mediated synaptic silencing (ΔfEPSP slope: −4.1% ± 6.0%; n = 8; one-way ANOVA: F = 0.392, p = 0.681; unpaired two-tailed t test, HEPES control versus pH clamped: p = 0.002; Figure 3D). These findings, together with the two-photon pH-imaging results above, support a mechanism for silencing of synaptic transmission that is mediated by an increase in pH restricted to ArchT3.0-expressing boutons.

### Trial-Limited Archaerhodopsin-Mediated Silencing of Synaptic Transmission Reveals a Differential Requirement of Left and Right CA3-CA1 Synapses during Long-Term Memory Acquisition

**Figure 4:**
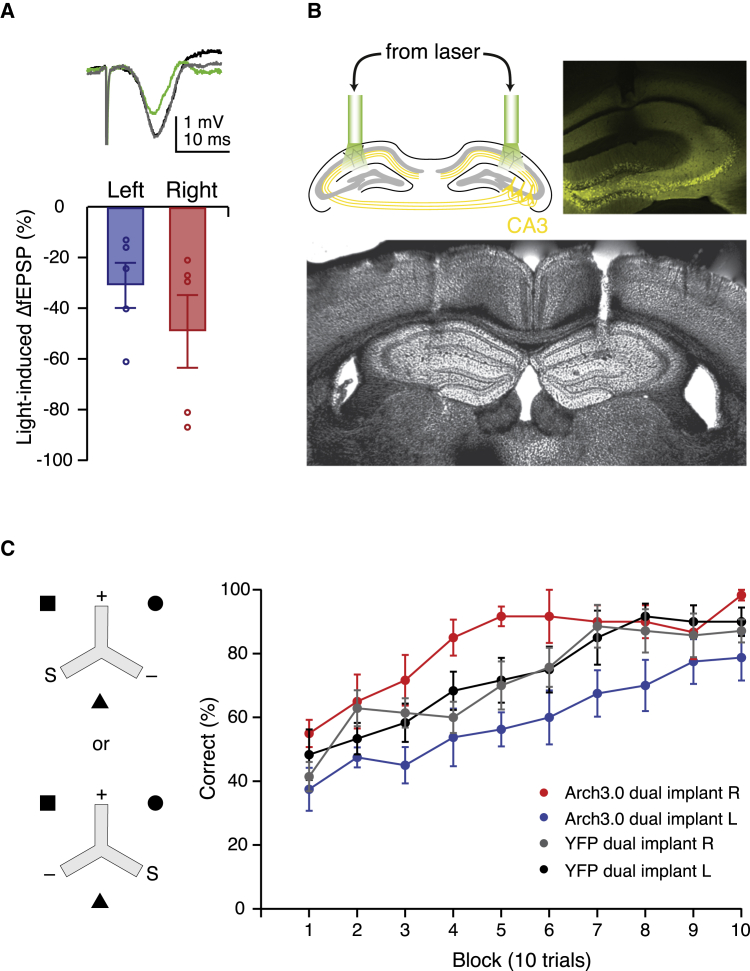
Optogenetic Synaptic Silencing Reveals a Unique Requirement for CA3-CA1 Synapses with Afferents Originating from Left, but Not Right, CA3 for Performance on the Y-Maze Reference Memory Task (A) In vivo fEPSP recordings in Grik4-Cre mice injected with a Cre-dependent Arch3.0 construct in the CA3: light-induced (532 nm, 30 mW for 2 min) change in fEPSP slope was robust and not significantly different between left and right CA3-CA1 synapses. Inset: representative fEPSPs before (black), during (green), and after (gray) light delivery. (B) Dual implant setup for synaptic silencing. Top left: two optical cannulae were surgically inserted above the CA1 in each hemisphere and later connected to a high-power 532-nm laser for light delivery. Top right: representative native eYFP fluorescence in a 60-μm coronal slice from a CA3-injected Grik4-Cre mouse. Bottom: representative bright-field image of a 60-μm coronal slice showing tracks made by dual implants above the CA1. (C) Left: mice were placed on an elevated Y-maze and had to use extra-maze cues to locate reward in one of the three arms. Mice started from the arm to the left of the rewarded arm in 50% of the trials and from that to the right in the other 50%, in a pseudorandomized order. S, start arm; +, rewarded arm; −, unrewarded arm. Right: light delivery to left Arch3.0-eYFP-expressing (blue), but not right Arch3.0-eYFP-expressing, CA3-CA1 afferents (red) impairs learning of the Y-maze reference memory task relative to the corresponding eYFP-only controls (left = black; right = gray). Error bars represent mean ± SEM. See also Figures S2 and S3.

To assess the utility of archaerhodopsin-mediated silencing of synaptic transmission in awake, behaving animals, we used it to investigate the roles of distinct, but spatially intermixed, subgroups of CA3-CA1 inputs during hippocampus-dependent learning. While mouse CA1 pyramidal neurons receive extensive CA3 afferents from both the ipsilateral and contralateral hemisphere (Amaral and Lavenex, 2007), previous findings have revealed distinct molecular and physiological properties of synapses onto CA1 neurons made by left CA3 afferents compared to those from the right CA3 (Kawakami et al., 2003, Shinohara et al., 2008, Kohl et al., 2011, Shipton et al., 2014; reviewed in El-Gaby et al., 2015). Furthermore, we have recently demonstrated a requirement for left, but not right, CA3 pyramidal neurons for hippocampus-dependent long-term memory (Shipton et al., 2014). We therefore hypothesized that CA3-CA1 synapses originating from the left and those from the right hemisphere could be differentially required during hippocampus-dependent learning (El-Gaby et al., 2015). To test this, we stereotactically injected an adeno-associated viral construct encoding either Cre-dependent Arch3.0-eYFP (test mice) or Cre-dependent eYFP (control mice) under the control of the elongation factor 1 alpha (EF1α) promoter (Mattis et al., 2011) unilaterally into either the left or right CA3 of Grik4-cre mice, which express Cre-recombinase selectively in CA3 neurons (Nakazawa et al., 2002). This enabled us to functionally dissociate the CA3-CA1 synapses with afferents originating from the left or right hemisphere. First, to confirm that Arch3.0 was capable of synaptic silencing in Grik4-cre mice, we recorded fEPSPs in vivo (Figure 4A). These recordings revealed strong Arch3.0-mediated silencing of synaptic transmission and no significant differences between silencing left and right CA3-CA1 synapses (ΔfEPSP slope: left −31.0% ± 8.9% [n = 5], right −49.1% ± 12.4% [n = 5]; unpaired two-tailed t test, left versus right: p = 0.312; Figure 4A). For behavioral testing, Grik4-cre mice that had been unilaterally injected with Cre-dependent Arch3.0-eYFP in the left or right CA3 also had fiber optic cannulas implanted bilaterally above the CA1 to silence both ipsilateral (Schaffer collateral) and contralateral (commissural) Arch3.0-expressing CA3-CA1 projections (Figure 4B). We subsequently employed a hippocampus-dependent, appetitively motivated reference memory task (Shipton et al., 2014) in which mice were given 10 blocks of 10 trials to learn to locate a reward in 1 of 3 arms of an elevated Y-maze using extra-maze cues (Figure 4C). To achieve maximal silencing of synaptic transmission, light was delivered for a period of 1 min before and for the entire duration of each trial. Control eYFP injected-only Grik4-cre mice were subjected to the same implant surgery and light delivery as Arch3.0-injected test subjects. We found that silencing CA3-CA1 synapses originating from the left, but not right, hemisphere significantly impaired task performance relative to eYFP controls (left-Arch3.0: n = 8 mice; right-Arch3.0: n = 6 mice; left-YFP: n = 6 mice; right-YFP: n = 7 mice; two-way ANOVA: no main effect of transgene [Arch3.0/YFP]: F = 0.193, p = 0.664; a main effect of hemisphere: F = 8.60, p = 0.007; a transgene by hemisphere interaction: F = 10.53, p = 0.004; analysis of simple main effects showed a significant effect of transgene on the left hemisphere [F = 7.00, p = 0.014] and a significant effect of hemisphere for Arch3.0 [F = 19.7, p < 0.001]; Figure 4C). There were no differences in implant placement between all groups in all three spatial dimensions (Figure S2). Furthermore, there was no difference in performance between mice in which CA3-CA1 synapses originating from the left hemisphere were silenced and those that were subjected to silencing of left CA3 neurons (Figure S3). This finding suggests that the requirement of the left CA3 during Y-maze reference memory performance (Shipton et al., 2014) can be explained by a necessity for synaptic transmission between the left CA3 and its bilateral postsynaptic targets in the CA1. Thus, silencing of synaptic transmission with Arch3.0 revealed a unique requirement for CA3-CA1 synapses originating from the left CA3, but not those from the right CA3, for performance on a hippocampus-dependent long-term memory task.

## Discussion

The above results demonstrate that archaerhodopsins are capable of robust and reversible silencing of synaptic transmission with relatively rapid onset and offset. Surprisingly, our data reveal that a change in presynaptic pH, rather than hyperpolarization, is necessary for archaerhodopsin-mediated silencing of synaptic transmission. This change in pH likely acts to acutely block evoked neurotransmitter release at archaerhodopsin-expressing presynaptic boutons. Such an acute and synapse-selective silencing tool provides a high level of precision in addressing synaptic function in behaving animals, as exemplified by its use here to reveal a functional distinction among CA3-CA1 synapses during hippocampus-dependent learning.

### Archaerhodopsin Activation in Axons Produces pH-Mediated Silencing of Synaptic Transmission

Our data show that selective archaerhodopsin activation in axons is sufficient to acutely reduce fEPSPs. Since archaerhodopsin expression was targeted to CA3-CA1 afferents from one hemisphere while fEPSPs were recorded from afferents originating from both hemispheres, the ∼50% maximal reduction observed suggests a maximal or near maximal silencing of archaerhodopsin-expressing inputs. Archaerhodopsins have been assumed to achieve neuronal silencing predominantly via hyperpolarization with minimal contribution from pH changes (Chow et al., 2010). Here, we show that in presynaptic boutons—which have a high plasma-membrane SAV ratio compared to neuronal somata—large, transient, and localized alkalinization of intracellular pH is induced by archaerhodopsin activation. Crucially, we demonstrate that archaerhodopsin-mediated pH changes are necessary for the reduction of fEPSPs.

Three findings indicate that archaerhodopsin reduced fEPSPs by direct effects on presynaptic neurotransmitter release without blocking action potential propagation. First, archaerhodopsin activation under our conditions robustly reduced fEPSPs without affecting the presynaptic fiber volley, which reflects the action potentials in the afferent fibers (Figure 1F). Second, the time course of archaerhodopsin-mediated fEPSP reduction (Figure 1) was slower than that of its hyperpolarizing current, which has an onset on a millisecond timescale (Chow et al., 2010, Han et al., 2011; Figure 3B). A similarly slow time course of archaerhodopsin effect on evoked synaptic transmission was observed by Mahn et al. (2016). Third, fEPSP reduction by archaerhodopsin was abolished by isosmotic addition of TMA and propionate, a manipulation that reduces pH changes, but does not reduce archaerhodopsin-mediated hyperpolarizing currents (Figures 3B and 3D). The current measurements were made at neuronal somata rather than at boutons; nevertheless, proton availability, which should be more limiting for proton pump activity at smaller compartments, would be expected to be enhanced by TMA and propionate since they act as open system buffers at a local level. Consequently, the presence of TMA and propionate would be expected to increase ArchT3.0-mediated hyperpolarizing currents at boutons, thus providing additional support for the notion that the blockade of synaptic silencing in the presence of these buffers is due to their blockade of pH changes (Figure 3C) rather than a reduction in ArchT3.0-mediated currents. Several candidate mechanisms could explain how an increase in bouton pH could interfere with neurotransmitter release, including reduced proton gradients across synaptic vesicles, deprotonation of calcium binding sites on the vesicular calcium sensor synaptotagmin, and/or deprotonation of constituents of the SNARE complex, temporarily preventing proper assembly for vesicle fusion and transmitter release (Chesler, 2003, Caldwell et al., 2013, Sinning and Hübner, 2013).

The pH dependence uncovered here suggests that the efficacy of archaerhodopsin as a silencer of synaptic transmission will depend on the SAV ratio, pH buffering capacity, and proton availability for a given axon and/or bouton(s). These factors may differ between different populations of axons/boutons (e.g., due to distinct axon morphologies; Pantazis et al., 2006), and even within the same group of axons/boutons due to distinct physiological (e.g., activity levels; Willoughby and Schwiening, 2002) or pathological states (e.g., acidosis due to ischemia; Meyer et al., 1986). While the level of archaerhodopsin-mediated silencing was not significantly different between the two hemispheric inputs compared in this study, comparisons between other synapses may be confounded by systematic differences in archaerhodopsin-mediated pH changes. Moreover, extra-bouton effects of archaerhodopsin activation could potentially contribute to the synaptic silencing effect, for example, via acidification of the synaptic cleft (Palmer et al., 2003). However, the behavioral data presented here are consistent with archaerhodopsin-mediated silencing exhibiting a high degree of synapse selectivity. Nevertheless, future applications of light-driven proton pumps as silencers of synaptic transmission should be carefully designed to take into account the mechanism underlying their effect and any accompanying physiological changes. For example, a recent study found that Arch3.0 activation at synaptic terminals causes a pH-mediated increase in intra-bouton calcium and a concomitant increase in the rate of spontaneous excitatory postsynaptic currents (EPSCs), both of which were independent of action-potential firing (Mahn et al., 2016). Conversely, Mahn et al. demonstrate a reduction in evoked EPSC amplitudes in response to the activation of Arch3.0 at thalamocortical terminals in acute slices, consistent with our data showing a reduction in the slope of evoked fEPSPs due to the activation of Arch3.0 and ArchT3.0 at CA3-CA1 synapses both ex vivo and in vivo. The discrepancy between the effect of proton pumps on spontaneous and evoked synaptic events may arise from differences between the synaptic release mechanisms underlying these two forms of synaptic events (reviewed in Ramirez and Kavalali, 2011). In particular, evoked release has a steeper dependence on calcium concentrations than that of spontaneous release, which may explain the robust ArchT3.0-mediated silencing of the former despite an increase in bouton calcium (Ramirez and Kavalali, 2011). Overall, the importance of pH highlighted here emphasizes the need for a more detailed consideration of the specific physiological changes mediated by optogenetic tools within defined subcellular compartments.

### The Spatiotemporal Precision of Archaerhodopsin-Mediated Silencing of Synaptic Transmission Enables Links between Synapse Activity and Behavior

The use of archaerhodopsin-mediated silencing of synaptic transmission has two important advantages. First, the silencing is highly spatially selective, with pH changes restricted to only those boutons expressing archaerhodopsin and no effect on action potential propagation; this permits high spatial precision of synaptic silencing within the expressing projection region exposed to light, while sparing axons of passage.

Second, the onset and offset of the archaerhodopsin-mediated synaptic silencing effect occur within seconds, making it markedly faster than previously used synaptic silencers (Stachniak et al., 2014, Lin et al., 2013). This makes archaerhodopsin a useful tool for trial-limited interference with synaptic transmission during behavioral testing, especially when multiple trials are necessary. It is possible that archaerhodopsin activation at other synapses and/or using other expression vectors could give rise to quantitative or qualitative differences in the time course of reduction in synaptic transmission as a result of differences in the biophysical and/or morphological properties of such synapses or due to differences in the level of archaerhodopsin expression and/or activation. For example, archaerhodopsin activation at smaller boutons with a larger SAV ratio would produce increases in pH more rapidly and hence cause faster onset of synaptic silencing. On the other hand, it is possible that large boutons with a lower SAV ratio may enable silencing via hyperpolarization-mediated block of action potential conduction because the current generated would not be limited by the proton gradient that builds. Even faster synaptic silencers will nevertheless be necessary in order to interrogate network dynamics on a millisecond timescale.

We demonstrate the use of archaerhodopsin-mediated synaptic silencing in a trial-limited manner to uncover a functional dissociation between two subsets of CA3-CA1 synapses during learning. Specifically, we find that CA3-CA1 synapses originating from the left hemisphere, which exhibit robust long-term potentiation (Kohl et al., 2011, Shipton et al., 2014), are necessary for hippocampus-dependent long-term memory performance; in contrast, stable CA3-CA1 synapses originating from the right hemisphere (Kohl et al., 2011, Shipton et al., 2014) are not required. This opens a unique window into investigating the function of plastic versus stable synapses for memory processing within the hippocampal network. Previous studies have used large-scale, long-lasting manipulations such as hippocampus-wide lesions and/or pharmacological inhibition to investigate hemispheric asymmetries in the rodent hippocampus and have found no clear evidence for such an asymmetry (e.g., Fenton and Bures, 1993, Li et al., 1999, Gerlai et al., 2002). Furthermore, tetanus-toxin based chronic inhibition of all CA3 output in mice does not impair learning on an incremental hippocampus-dependent long-term memory task (Nakashiba et al., 2008). However, these manipulations likely recruit compensatory changes that allow use of alternative circuits (e.g., the hippocampal circuit in the opposite hemisphere) or behavioral strategies to solve a given task (Turrigiano, 2008, Goshen et al., 2011). The trial- and synapse-limited silencing achieved here makes it unlikely that homeostatic changes will alter task performance and hence enables more direct links to be established between synapse properties, circuit function, and behavior.

## Experimental Procedures

### Viral and Implant Surgery

Male C57BL/6J mice (Charles River Laboratories) received single or dual stereotactic injections of pAAV5-CaMKIIa-eArcht3.0-EYFP or pAAV5-CaMKIIa-EYFP constructs in either the left or right CA3 or CA1. Grik4-Cre mice (Nakazawa et al., 2002) received dual stereotactic injections of AAV5-Ef1a-DIO-eArch3.0-eYFP or AAV5-Ef1a-DIO-eYFP constructs in either the left or right CA3. For behavioral synaptic silencing experiments, two fiber optical cannulas were implanted above the CA1 of each hemisphere in Grik4-cre mice that had received dual stereotactic injections of AAV5-Ef1a-DIO-eArch3.0-eYFP or AAV5-Ef1a-DIO-eYFP constructs in either the left or right CA3. All experiments were performed in accordance with U.K. Home Office Regulations and under personal and project licenses held by the authors.

### Electrophysiological Protocols and Light Delivery

#### Extracellular Recordings

Both ex vivo and in vivo extracellular field recordings were made in the CA1 of mice expressing either ArchT3.0-eYFP or eYFP in either left or right CA3 neurons and their projections.

For ex vivo field excitatory post synaptic potential (fEPSP) recordings, slices were transferred to an interface-style recording chamber maintained at 31°C–33°C superfused with carbogen-bubbled bicarbonate-based ACSF (as used during slicing; see Supplemental Experimental Procedures) at a rate of 0.5 ml min^−1^. Recording started at least 10 min after the slices were transferred. For some experiments (see main text), slices were instead superfused with one of two HEPES-based ACSF solutions during recordings, as follows.

(1) Control HEPES solution contained 145 mM NaCl, 3 mM KCl, 15 mM HEPES, 2 mM CaCl_2_, 10 mM glucose, and 2 mM MgCl_2_ (Figures 3B–3D).
(2) The “pH clamping” solution contained 125 mM NaCl, 20 mM Na(C_2_H_5_COO), 3 mM (CH_3_)_3_N·HCl, 3 mM KCl, 15 mM HEPES, 2 mM CaCl_2_, 10 mM glucose, and 2 MgCl_2_ (Figures 3B–3D).

All solutions were brought up to a pH of 7.40 by adding NaOH and bubbled with 100% O_2_ gas. Experiments in these HEPES-based solutions were carried out with slices from mice injected at two sites in the CA3 of the same hemisphere. All other conditions were identical to those under bicarbonate-based ACSF.

Recordings were made with an Axoclamp-2A amplifier in bridge mode and data acquired with an Instrutech ITC-16 A/D board (Instrutech) using Igor Pro software (WaveMetrics). Synaptic efficacy ex vivo was monitored by stimulating the Schaffer collaterals at 0.2 Hz (50 μs, 30–300 μA) with a 2-MΩ monopolar tungsten electrode (A-M Systems) connected to a stimulus isolator unit (ISO-flex, A.M.P.I.). Recordings were performed with glass pipettes (3 to 8 MΩ) made from standard borosilicate glass capillaries (outer Ø: 1.2 mm, inner Ø: 0.68 mm; World Precision Instruments) using a horizontal P-97 pipette puller (Sutter Instruments). The glass pipettes were filled with ACSF and placed in the CA1 stratum radiatum, distal to the stimulating electrode. Light was delivered using a 200-μm diameter, 0.39-NA optical fiber (Thorlabs) connected to a green (532-nm) fiber-coupled high-power laser (Thorlabs) also placed in the stratum radiatum, as close as possible to the stimulation electrode to ensure maximal overlap of fibers recruited by electrical stimulation and those targeted by light. Stimulation strength was set to elicit a fEPSP of ∼1 mV. fEPSP slopes were monitored for a baseline period of at least 2 min. If synaptic transmission was stable (<10% change in fEPSP slopes over 2 min), light was delivered for a duration of 2 min at an intensity of 2 mW. Recordings continued for at least another 5 min following light delivery.

For in vivo recordings, C57BL/6J mice that had received a single CA3 injection of ArchT3.0-eYFP or eYFP construct and Grik4-cre mice that had received two injections in the same hemisphere of an Arch3.0-eYFP construct were anaesthetized by intraperitoneal injection of ∼1.5 g kg^−1^ urethane delivered over at least four separate injections. Rectal temperature was kept at 36°C–37°C by means of a heating pad placed beneath the animal. ACSF was applied to the skull and recording, and stimulating grounds were placed beneath the skin surrounding the skull. Using a stereotactic apparatus (Kopf Instruments), a small craniotomy was made 2.4, 1.94, or 1.70 mm posterior and 1.5, 1.25, or 0.8 mm lateral from bregma, respectively. In ArchT3.0-eYFP construct-expressing mice, these were done contralateral to the injection side, to ensure no contamination from somatic ArchT3.0 effects. A 1 MΩ monopolar tungsten stimulating electrode (A-M Systems), 200-μm diameter, 0.39-NA optical fiber and ACSF-filled, borosilicate glass recording electrode were attached together and lowered onto the CA1 through a durotomy to reach 1.4–1.7 mm below bregma. Light was delivered at 10, 15, 20, 25, or 30 mW (in a randomized order) for 2 min each time. To confirm that recordings were carried out in the CA1, at the end of all recording sessions with a particular mouse, a 2-s DC current was passed at each of the recording sites, causing small lesions that were inspected through a bright-field microscope. All other conditions were identical to those for the ex vivo recordings described above.

Analysis was performed using Igor Pro (WaveMetrics). Changes in synaptic efficacy were estimated using the mean fEPSP slopes (middle third of rising slope) from the second minute of light delivery (when the synaptic silencing effect has plateaued), normalized to the mean fEPSP slope during the last minute of baseline recording. Given that there were no differences in the magnitudes of light effect as a result of injected hemisphere or site of recording relative to injected hemisphere (i.e., slices ipsilateral or contralateral to injected hemisphere), all results were pooled (see below for statistical analysis). Silencing and recovery time constants were calculated by fitting exponential functions to the normalized fEPSP slopes during light application and post-light recovery, respectively. The reversibility of ArchT3.0-mediated fEPSP reduction was established both in vivo and ex vivo in bicarbonate-based ACSF. Subsequently, for mechanistic dissection in the HEPES-based ACSF solutions, experiments in which the fEPSP did not recover post-light were not included.

#### Intracellular Recordings

For voltage-clamp experiments, slices from mice expressing ArchT3.0-eYFP in CA1 neurons were transferred to a submerged-style recording chamber and superfused with HEPES-based ACSF at a rate of 1–2 ml min^−1^. Control and “pH clamp” solutions were added sequentially in a randomized order for each cell, with at least a 20 min perfusion time for each solution before recordings were made. Whole-cell voltage-clamp recordings were performed with borosilicate glass pipettes (3 to 5 MΩ). A cesium-methylsulphonate-based intra-pipette solution was used containing 120 mM CsCH_3_SO_3_, 20 mM CsCl, 0.2 mM EGTA, 10 mM HEPES, 10 mM QX-314, 4 mM Mg-ATP, and 0.3 mM GTP. Final pH was 7.2–7.3, and final osmolarity was 285 to 300 mOsm L^−1^.

Cells with a pyramidal-shaped soma in the stratum pyramidale of CA1 were selected for recording using infrared, differential interference contrast optics. To allow diffusion of Cs^+^ into the dendrites for improved space clamp, voltage-clamp recordings were not started until at least 10 min after entering whole-cell mode. Series resistance was not corrected for but was monitored continuously during recordings; recordings were rejected if the series resistance changed by more than 25%. ArchT3.0-mediated currents were elicited by delivering a 100-ms pulse of 2 mW light using a 200-μm diameter, 0.39 NA optical fiber connected to a green (532 nm) fiber-coupled high-power laser. Data were low-pass filtered at 2 kHz and acquired at 20 kHz with an AxonMulticlamp700B amplifier (Molecular Devices) using custom software (MatDAQ, Hugh P.C. Robinson 1995–2013) programmed in MATLAB (MathWorks). The maximal current for each trace was recorded, and the mean of these maximal values from at least five traces was calculated for each cell in each condition.

### pH Imaging

Images were obtained from slices expressing ArchT3.0-eYFP in CA3 neurons and their projections that had been pre-incubated with HPTS (see above) using a high-resolution, two-photon imaging microscope (Intelligent Imaging Innovations). Slices were transferred to an interface-style recording chamber maintained at 31°C–33°C, superfused with carbogen-bubbled bicarbonate-based or oxygen-bubbled HEPES-based ACSF at a rate of 1–2 ml min^−1^. For imaging, we used femtosecond laser pulses from a Ti:sapphire laser (MaiTai DeepSee; Spectra-Physics), coupled to a galvanometer scanning unit (3i Vector) that was mounted on a modified Zeiss AxioExaminer Z1 upright microscope equipped with a 20×/1.0 NA objective. Fluorescence was acquired using non-descanned GaAsP detectors. All images were acquired using a 525 nm filter (40 nm bandwidth). Expression of eYFP was detected by two-photon illumination at 980 nm (2.5 mW), a wavelength at which HPTS fluorescence was not detectable under our conditions. Recordings were also made with two-photon illumination at 800 nm (10 mW), a wavelength at which HPTS, but not eYFP, fluorescence was detected. Thus, eYFP expression was estimated by measuring the ratio of fluorescence at 980 and 800 nm, with the HPTS fluorescence reading at 800 nm serving as a within-bouton or process or soma control. We found no significant difference in this estimate of ArchT3.0-eYFP expression levels between boutons and soma (980/800 ratio boutons: 0.34 ± 0.08; 980/800 ratio soma: 0.15 ± 0.13; unpaired two-tailed t test: p = 0.167). Ratiometric recordings of pH were carried out by two-photon illumination first at 900 nm, then at 750 nm, both at 10 mW, and calculating the fluorescence intensity ratio at these wavelengths, measured using ImageJ software. The 900/750 ratio was highly sensitive to pH changes around pH 7–8 (Figure 2A).

When investigating pH changes at boutons, imaging was carried out in the CA1 stratum radiatum of slices contralateral to the viral injection site to ensure that any fluorescence detected was in the axons and/or boutons of CA3 projections. To activate axonal ArchT3.0, light was delivered using a 200-μm diameter, 0.39-NA optical fiber (Thorlabs) connected to a green (532-nm) fiber-coupled high-power laser (Thorlabs) at 2 mW for 2 min to mimic conditions during fEPSP reduction. Ratiometric 900/750 measurements were taken from images at the following three time points: (1) before light delivery, (2) immediately after light cessation (a time at which fEPSPs are still effectively silenced, but no contamination from laser light is present; Figures 1E and 1F), and (3) 6 min after light cessation (a time at which fEPSPs have completely recovered from ArchT3.0-mediated silencing; Figures 1E and 1F). Five vertical stacks of images (resolution 512 × 256 pixels per frame), separated by 1 μm, were taken at each time point. ArchT3.0-eYFP-expressing boutons were identified as round processes with a diameter of ∼1–2 μm that showed eYFP fluorescence at 980 nm. Similarly sized processes showing no ArchT3.0-eYFP fluorescence were used as within-slice controls. CA3 boutons in the contralateral CA1 from eYFP-only injected mice were subjected to the same experimental procedure and served as additional controls. In addition, identical conditions were used to obtain pH measurements from ArchT3.0-expressing CA3 somata in slices ipsilateral to the viral injection site. 900/750 fluorescence ratios were used to estimate pH values from a sigmoidal function fit to an experimentally determined calibration curve (Figure 2A).

### Behavioral Experiments

#### Y-Maze Long-Term Memory Task

Following a pre-training period (see the Supplemental Experimental Procedures), food-restricted mice were assigned a rewarded target arm in an elevated Y-maze. Target arm designations were counterbalanced such that approximately equal proportions of each experimental group were assigned to each arm. Mice were started facing outward in either the left or right arm relative to the target arm and received 10 trials per day for 10 consecutive days. On each day, mice had five starts from the left of the target arm and five starts from the right in a pseudorandom order with no more than three consecutive starts from the left or right. Mice were started in a pseudorandom order within the cage that varied across trials, and the inter-trial interval (ITI) was approximately 15 min. Green laser light from a solid-state laser diode (532 nm, 24 ± 3 mW at fiber tip, Laser 2000) was collimated and split into two aperture-matched fiber optic patch cords (Doric Lenses) that were connected to the two fiber optic implants. Connection of the implant to the laser and illumination began before each animal was placed on the maze. To ensure maximal synaptic silencing was reached during each trial, light was on for 1 min before the trial started and then continuously for the duration of the trial and was turned off once the mouse was removed from the maze. If a mouse made the correct choice, it was allowed to consume the reward. To prevent arm re-entry errors that could otherwise provide an additional source of learning impairment, the trial was ended if mice chose the incorrect arm and after they had seen the empty food well. The maze was pseudorandomly rotated either clockwise or anticlockwise between trials to ensure that olfactory, tactile, or visual cues on the maze itself did not provide information that could be used to solve the task. On the last day of testing, food was only delivered once the mouse reached the food well to check that mice did not use reward odor to solve the task. When not performing the task, mice were kept behind a screen to minimize exposure to the testing room and cues in the absence of laser light.

### Statistical Analysis

Statistical analysis was performed using the R software as described in the main text. All values are given as mean ± SEM.

Numbers (n) refer to the number of animals for behavioral measurements, number of recording sites for in vivo fEPSP recordings, number of slices for ex vivo fEPSP recordings, number of cells for intracellular recordings, and number of cells, ArchT3.0-eYFP- or eYFP-only, expressing boutons or “control” processes (as specified in text) for pH imaging experiments.

For more detailed descriptions, see the Supplemental Experimental Procedures.

## Author Contributions

M.E., C.J.S., O.P., and O.A.S. designed the experiments. M.E. and O.A.S. performed surgeries. M.E. and K.W. performed electrophysiological experiments. M.E. and Y.Z. performed imaging experiments. M.E. performed behavioral experiments. M.E., Y.Z., C.J.S, O.P., and O.A.S. analyzed the data and wrote the manuscript. All authors approved the manuscript.

## Supplemental Information

Document S1. Supplemental Experimental Procedures and Figures S1–S3

Document S2. Article plus Supplemental Information
