# Supplementary material for: Archaerhodopsin Selectively and Reversibly Silences Synaptic Transmission through Altered pH
Source: Cell Rep. 2016 Aug 11;16(8):2259–68. doi: 10.1016/j.celrep.2016.07.057 (PMC4999416; doi:10.1016/j.celrep.2016.07.057)
Supplement: Document S1. Supplemental Experimental Procedures and Figures S1–S3 [file mmc1.pdf]

Cell Reports, Volume *16*

Supplemental Information

Archaerhodopsin Selectively and Reversibly

Silences Synaptic Transmission through Altered pH

Mohamady El-Gaby, Yu Zhang, Konstantin Wolf, Christof J. Schwiening, Ole Paulsen, and Olivia A. Shipton

Supplemental Experimental Procedures

Animals

All experiments were performed in accordance with U.K. Home Office Regulations and under personal and project licenses held by the authors. Male C57BL/6J mice (Charles River Laboratories) and Grik4-cre mice (Nakazawa et al., 2002; The Jackson Laboratory) were housed in polycarbonate cages of 5-10 mice on a 12-hour light-dark cycle, and had access to food and water ad libitum unless otherwise stated.

Virus injections for electrophysiology and 2-photon imaging

For recordings and imaging in wild-type C57BL/6J mice, a construct with ArchT3.0 fused in-frame to enhanced yellow fluorescent protein (eYFP) and driven by a CaMKIIα promoter was used (Mattis et al., 2011). For *in vivo* recordings in Grik4-Cre mice, a Cre-dependent construct with Arch3.0 fused in-frame to eYFP and driven by a EF1α promoter was used (Mattis et al., 2011). Adeno-associated viral particles of serotype 5 were produced by the Vector Core Facility at The University of North Carolina at Chapel Hill. Mice (13-16 weeks old) were anesthetized with 2-4% isoflurane at 0.6-1.4 L min^{-1}. Using a stereotactic apparatus (Kopf Instruments, Tujunga, CA), a small craniotomy was made 2.3 mm posterior and 2.2 mm lateral (either left or right) from bregma for single CA3 injections or 1.94 mm posterior and 1.25 mm lateral from bregma for single CA1 injections. For dual CA3 injections, two small craniotomies were made, the first at 1.46 mm posterior and 1.25 mm lateral to bregma (site 1) and the second 2.46 mm posterior and 2.4 mm lateral to bregma (site 2). Through a small durotomy, 0.8 µl virus suspension (pAAV5-CaMKIIa-eArcht3.0-EYFP, or pAAV5-CaMKIIa-EYFP 4 x 10^{12} viral molecules mL^{-1}; University of North Carolina Vector Core) were delivered at a rate of 0.1 µL min^{-1}, 2.25 mm (single CA3 injections) or 1.5 mm (CA1) below the skull surface from bregma through a 33-gauge needle using a Hamilton Microliter syringe. Following a 7-minute wait after bolus injection, the needle was retracted by 0.2 mm and after another 7 minutes slowly retracted fully. For dual CA3 injections, virus was delivered at 2.00 mm and 2.30 mm below the skull surface at bregma at sites 1 and 2 respectively. The scalp incision was then sutured, and post-injection analgesic (0.03 mg kg^{-1} buprenorphine) was administered intraperitoneally to aid recovery. Similarly, adult male Grik4-Cre mice received dual injections of AAV5-Ef1a-DIO-eArch3.0-eYFP at sites 1 and 2, as described above.

To allow sufficient ArchT3.0 or Arch3.0 expression in neuronal somata and axons, mice were not used for recordings or imaging until at least 8 weeks after viral injection.

Slice preparation for electrophysiology and 2-photon imaging

For *ex vivo* extracellular and intracellular recordings, coronal hippocampal slices (350 µm) were prepared after decapitation under deep isoflurane induced anesthesia. Following dissection in ice cold artificial cerebrospinal fluid (ACSF) containing (mM): NaCl 126; KCl 3; NaH_2PO_4 1.25; $MgSO_4$ 2; $CaCl_2$ 2; $NaHCO_3$ 25; glucose 10; pH 7.2-7.4; bubbled with carbogen gas (95% O_2, 5% CO_2), slices were maintained at room temperature (22-25 °C) in a submerged-style holding chamber for at least one hour before recording/imaging.

For pH measurements, slicing was performed in ACSF with the additional presence of 0.5 mM 8-hydroxypyrene-1,3,6-trisulfonic acid (HPTS). In addition, the initial 20 minutes of post-slicing incubation was carried out in the presence of 0.5 mM HPTS-containing ACSF to promote the uptake of the pH-dye into neuronal processes. Subsequently, the HPTS-containing ACSF was gradually washed off with an ACSF-only solution and slices were incubated in the latter solution for the remainder of their recovery period.

Correction of ΔpH values for eYFP contribution

The F(900/750) measure was unaffected by background eYFP (Bouton F(900/750) for HPTS in absence of ArchT3.0-eYFP expression (HPTS only): 4.99 ± 0.75, F(900/750) for HPTS in boutons with ArchT3.0-eYFP (eYFP+HPTS): 4.87 ± 0.37. Unpaired two-tailed *t* test (HPTS only vs. eYFP+HPTS: *P* = 0.854). However, changes in fluorescence in response to pH changes during calibration were primarily mediated by an increase in fluorescence when (2-photon) illuminating at 900 nm, as opposed to decreases in fluorescence at 750 nm. Therefore, in addition to raw ΔpH values, a corrected ΔpH was calculated for ArchT3.0-YFP-expressing boutons and somata. Correction was carried out by first calculating corrected ΔF(900/750) values as follows:

$$\Delta F\left(\frac{900}{750}\right)(corrected) = \Delta F\left(\frac{900}{750}\right)(measured)\times\left(\left(1-\left(0.08\times\frac{F(eYFP750)}{F(total750)}\right)\right)/\left(1-\left(0.92\times\frac{F(eYFP900)}{F(total900)}\right)\right)\right)$$

F(eYFP900)/F(total900) and F(eYFP750)/F(total750) represent the fraction of baseline fluorescence due to eYFP expression at 900 nm and 750 nm (calculated from (1-F(HPTS)/F(total) for the respective wavelength), respectively. The numbers 0.92 and 0.08 represent the relative contribution of the increase in fluorescence at 900 nm and decrease in fluorescence at 750 nm, respectively, to changes in the 900/750 ratio around the measured baseline pH values. The corrected ΔF(900/750) values were then used to estimate corrected ΔpH values by addition to the baseline F(900/750) values to calculate corrected F(900/750)(after) values and hence estimating pH values after ArchT3.0 activation. The corrected values did not differ significantly from uncorrected values for all conditions (Bicarbonate ACSF: ΔpH boutons (uncorrected) = 0.82 ± 0.20, ΔpH boutons (corrected) = 0.87 ± 0.20; Paired two-tailed *t* test uncorrected vs. corrected: *P* = 0.055. ΔpH soma (uncorrected) = -0.02 ± 0.06, ΔpH soma (corrected) = 0.03 ± 0.08; Paired two-tailed *t* test uncorrected vs. corrected: *P* = 0.507. HEPES-Ctr ACSF: ΔpH boutons (uncorrected) = 0.89 ± 0.24, ΔpH boutons (corrected) = 0.84 ± 0.24; Paired two-tailed *t* test uncorrected vs. corrected: *P* = 0.355. 'pH-clamped' ACSF: ΔpH boutons (uncorrected) = -0.01 ± 0.07, ΔpH boutons (corrected) = 0.06 ± 0.08; Paired two-tailed *t* test uncorrected vs. corrected: *P* = 0.069).

Modelling effect of plasma membrane surface-area to volume ratio on ΔpH in soma and boutons

To model the effect of soma versus bouton differences in membrane surface-area to volume (SAV) ratio on the pH effect we assumed a similar plasma-membrane ArchT3.0 density and function at the soma and boutons, and hence similar proton flux per unit plasma membrane area. We also assumed similar pH buffering properties within the two compartments. We reconstructed 7 boutons from the imaged stacks. To achieve this, the cross sectional area in each plane in which the bouton was visible was measured and multiplied by 1 μm (the z-distance between each plane) and then added to give an estimate of the volume. Subsequently, membrane surface area was estimated by assuming a spherical shape and calculating a virtual radius from the estimated volume. The resulting mean surface-area to volume (SAV) ratio (4.2 ± 0.1 μm^{-1}, n = 7) was then compared to that expected for a sphere of 5 μm diameter (representing the minimum size accepted for a neuronal soma). Working from the estimated ΔpH for boutons, we used this, as well as the estimated baseline pH for both soma and boutons, to predict ΔpH for the soma as follows:

$$\frac{\Delta[H^+](bouton)}{\Delta[H^+](soma)} = \frac{SAV(bouton)}{SAV(soma)}$$

Subsequently, post-light $[H^+]$ at the soma was estimated by adding $\Delta[H^+]$(soma) to the baseline $[H^+]$ calculated from the measured baseline pH at the soma and subsequently estimating post-light pH and hence ΔpH.

To determine the relationship between bouton SAV ratio and pH changes, a value for SAV was estimated for all ArchT3.0-eYFP expressing boutons. SAVs were estimated (assuming spherical shape) from virtual radii calculated from measured bouton cross-sectional areas since not all boutons could be reconstructed (because parts of the bouton was outside the z-extent of 5 imaged stacks). The mean bouton SAV ratio estimated using this method was 6.2 ± 0.2 μm^{-1} (n = 18).

Behavioral experiments

Animals and AAV vectors

Male Grik4-cre mice (The Jackson Laboratory) and C57BL/6J mice (Charles River Laboratories, UK or Harlan, UK) were housed in polycarbonate cages with 5-10 mice in each on a 12-hour light-dark cycle, and had access to food and water ad libitum, except when on food restriction during appetitively-motivated behavioral tests.

Surgery and light delivery

For behavioral experiments, mice (9-36 weeks old) were anesthetized with 2-4% isoflurane at 0.6-1.4 L min^{-1}, placed on a heating pad to aid body temperature maintenance and their head fixed in a stereotactic apparatus (Kopf Instruments, Tujunga, CA). The head was levelled and three small craniotomies were made above either the left or right dorsal hippocampus. At sites 1 and 2 (1: anteroposterior [AP]: -1.46 mm, mediolateral [ML]: ±1.25 mm, dorsoventral [DV]: -2.00 mm; 2: AP: -2.46 mm, ML: ±2.40 mm, DV: -2.30 mm, all coordinates from skull surface at bregma) 0.75 µl virus suspension (AAV5-Ef1a-DIO-eArch3.0-eYFP, 4 x 1012 viral molecules mL^{-1} for test mice and AAV5-Ef1a-DIO-eYFP, 6 x 1012 viral molecules mL^{-1} for control mice; University of North Carolina Vector Core) were delivered at a rate of 0.1 µL min^{-1} through a 33-gauge needle using a Microliter syringe (Hamilton, UK). Following a 6-minute wait after bolus injection, the needle was retracted by 0.20 mm and after another 6 minutes wait slowly retracted fully. For single implanted mice, at site 3 (AP: -1.94 mm, ML: 2.00 mm, DV: -1.80 mm) a fiber optic cannula, 200 µm diameter, 0.37 NA (Doric Lenses,

Quebec, Canada) was placed above the left CA3 and secured to the skull using dental cement (C&B Metabond, Prestige Dental, UK and Simplex, Claudius Ash, UK). For dual implanted mice, at sites 3 (AP: -1.94 mm, ML: 1.25 mm, DV: -1.30 mm) and 4 (AP: -1.94 mm, ML: -1.25 mm, DV: -1.30 mm) a fiber optic cannula, 200 µm diameter, 0.37 NA (Doric Lenses, Quebec, Canada) was placed in each hemisphere and secured to the skull using dental cement (C&B Metabond, Prestige Dental, UK and Simplex, Claudius Ash, UK). The scalp incision was sutured and anti-inflammatory and analgesic drugs (2 mg kg^{-1} meloxicam; 0.1 mg kg^{-1} buprenorphine) were administered subcutaneously to aid recovery.

Y-maze long-term memory task: pre-training

Following at least 8 weeks for expression of eArch3.0-eYFP or eYFP to develop after surgery, mice were handled to habituate them to the experimenter and accustom them to the connection of the implant. All behavioral testing was done with the experimenter blind to condition.

The Y-maze was constructed of gray painted wooden arms (50 cm by 13 cm bordered by 1 cm high white plastic walls), extending from a central triangle. Metal food wells (1.5 cm high) were positioned 5 cm from the distal end of the arms. The maze was elevated 82 cm from the floor. Mice were put on a restricted feeding schedule, allowing them to maintain at least 85% of their free-feeding body weight. Mice were introduced to the food reward (0.1 mL of sweetened condensed milk diluted 50:50 with water) in their home cages to overcome neophobia and then pre-trained on the elevated Y-maze in a room different to where behavioral testing would occur until they were highly motivated to search for food and running freely on the Y-maze (reaching the food reward in under 30 s for three consecutive trials). Once these conditions were met, mice were then moved on to the test phase (see Experimental Procedures in main text).

Implant imaging

Mice were anesthetized by intraperitoneal injection of pentobarbital (533 mg kg^{-1}) and then transcardially perfused with cold phosphate-buffered saline (PBS, pH 7.4) followed by 4% (w/v) paraformaldehyde (PFA) in PBS. Brains were post-fixed for 36 hours at 4 °C in PFA in PBS, then rinsed and subsequently infiltrated with 30% (w/v) sucrose in PBS for at least 48 hours. Coronal sections of 60 µm thickness along the entire dorsoventral axis of the hippocampus were cut using a microtome (Spencer Lens Co., Buffalo, NY) and divided into three series. One series was used for implant scoring. Fluorescence (Zeiss Axioskop 2 microscope, Germany) and bright-field and fluorescence images were taken and scored blindly for implant location and native expression respectively.

Supplemental Figures

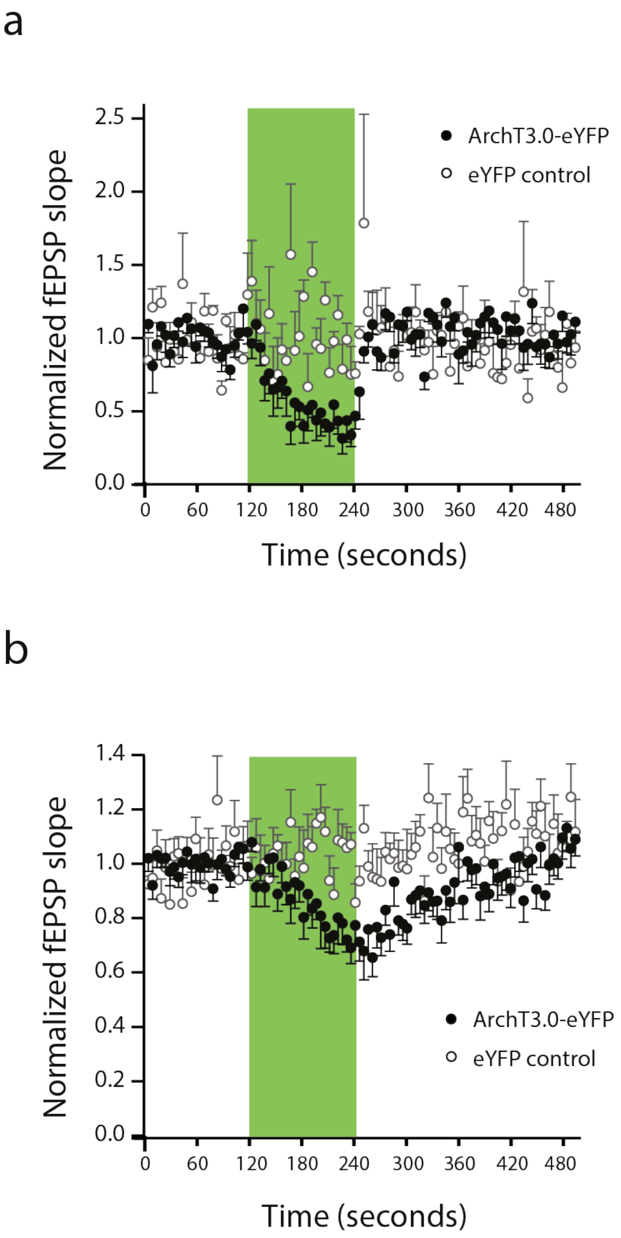

Figure S1 – Light delivery fails to reduce fEPSP slopes for eYFP-only expressing axons both *in vivo* and *ex vivo*

a) Green light (532 nm, 30 mW) delivered for 2 minutes *in vivo* caused a reversible reduction in the normalized fEPSP slope in ArchT3.0-eYFP expressing, but not in eYFP-only expressing, mice. Green area represents time of light delivery.

b) Green light (532 nm, 2 mW) delivered for 2 minutes *ex vivo* (in coronal slices) caused a reversible reduction in the normalized fEPSP slope in ArchT3.0-eYFP expressing, but not in eYFP-only expressing, mice. Green area represents time of light delivery.

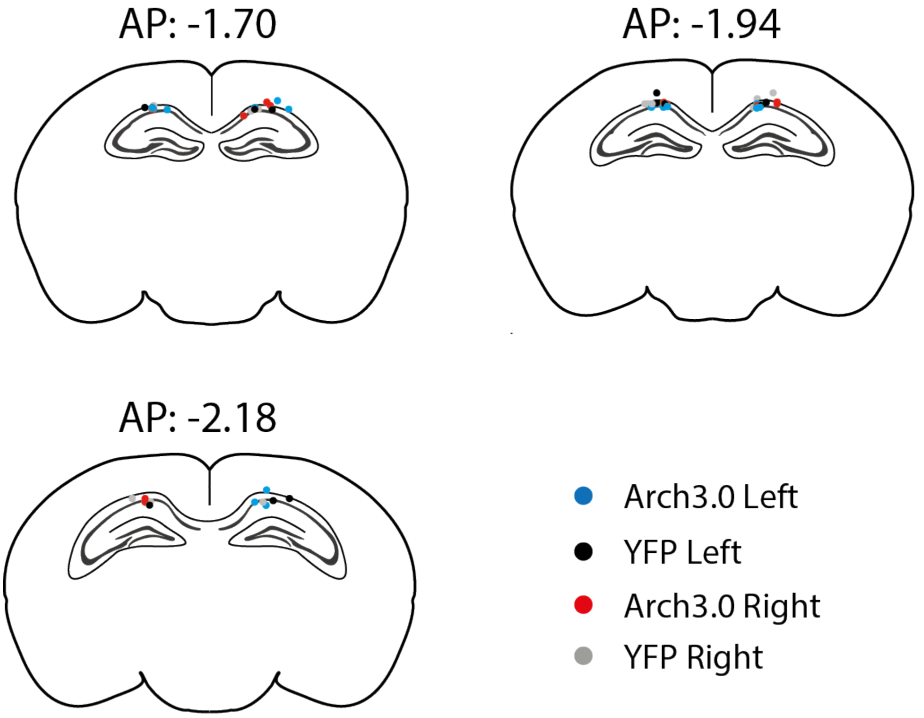

Figure S2 – Implant placements for synaptic silencing during behavior

Average position of the tip of each fiber optic implant (mean ± S.E.M):

Injected hemisphere/construct:

Left-Arch3.0 n= 8 mice, right-Arch3.0 n= 6 mice, left-YFP n= 6 mice, right-YFP n= 7 mice;

Left-Arch3.0 (ipsilateral): AP: -1.87 ± 0.06, ML: 1.18 ± 0.06, DV: -1.28 ± 0.02; Right-Arch3.0 (ipsilateral): AP: -1.82 ± 0.08, ML: 1.19 ± 0.11, DV: -1.24 ± 0.04; Left-YFP (ipsilateral): AP: -1.91 ± 0.08, ML: 1.23 ± 0.05, DV: -1.23 ± 0.05; Right-YFP (ipsilateral): AP: -1.88 ± 0.10, ML: 1.07 ± 0.08, DV: -1.26 ± 0.04.

Left-Arch3.0 (contralateral): AP: -1.99 ± 0.07, ML: 1.17 ± 0.09, DV: -1.27 ± 0.04; Right-Arch3.0 (contralateral): AP: -1.97 ± 0.07, ML: 1.22 ± 0.07, DV: -1.26 ± 0.02; Left-YFP (contralateral): AP: -1.96 ± 0.09, ML: 1.29 ± 0.11, DV: -1.26 ± 0.02; Right-YFP (contralateral): AP: -1.94 ± 0.12, ML: 1.41 ± 0.06, DV: -1.20 ± 0.05.

No significant differences in the average optical fiber placement in any spatial dimension between behavioral groups and between contralateral and ipsilateral implants (referred to as implanted-hemisphere). Multi-way, repeated-measures ANOVA:

Anteroposterior: no main effect of transgene $F = 0.014$, $P = 0.905$; no main effect of implant-hemisphere $F = 2.575$, $P = 0.115$; no main effect of injected-hemisphere $F = 0.188$, $P = 0.666$; and no transgene by injected-hemisphere interaction $F = 0.015$, $P = 0.904$.

Mediolateral: no main effect of transgene $F = 1.25$, $P = 0.269$; no main effect of implant-hemisphere $F = 3.36$, $P = 0.073$; no main effect of injected-hemisphere $F = 0.009$, $P = 0.925$; and no transgene by injected-hemisphere interaction $F = 0.237$, $P = 0.628$.

Dorsoventral: no main effect of transgene $F = 0.742$, $P = 0.394$; no main effect of implanted-hemisphere $F = 0.018$, $P = 0.893$; no main effect of injected-hemisphere $F = 0.505$, $P = 0.481$; and no transgene by injected-hemisphere interaction $F = 0.007$, $P = 0.934$.

All mice satisfied the criteria of eYFP expression in the CA3 and its axons and the ventral most tip of the implants being less than 0.1 mm from the target subfield.

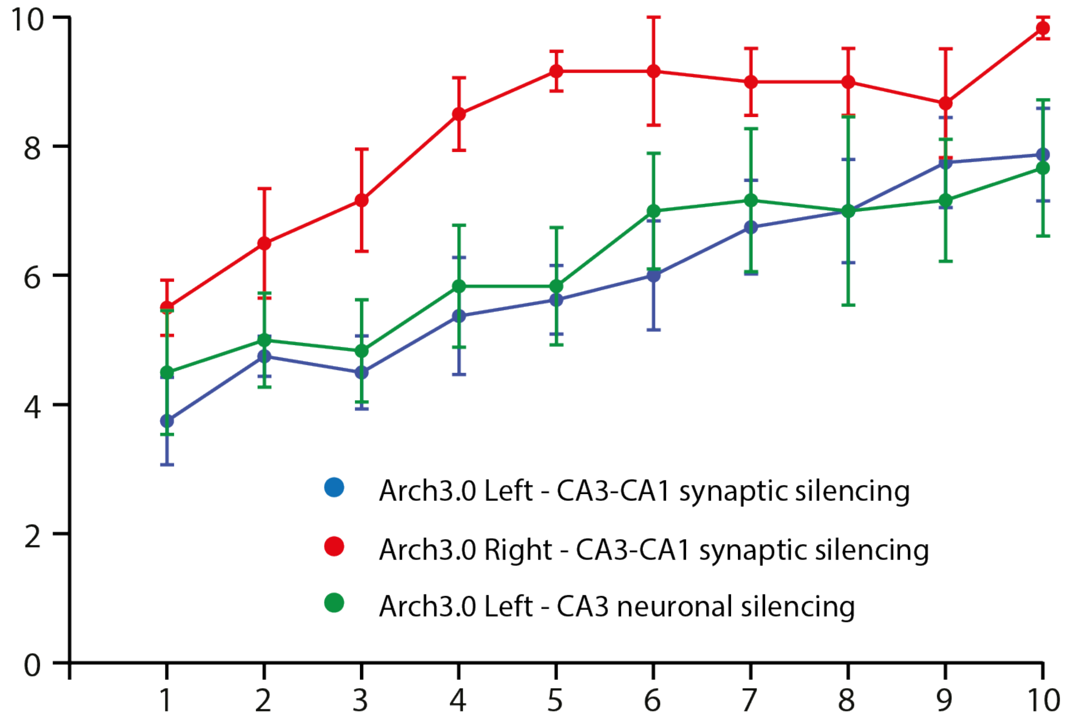

Figure S3 – Effect of neuronal silencing at left CA3 and synaptic silencing at left or right CA3-CA1 synapses on Y-maze performance

The performance of mice experiencing light delivery to left Arch3.0-eYFP-expressing CA3-CA1 afferents (left CA3-CA1 silencing; blue) and that of mice with light delivery to Arch3.0-eYFP-expressing CA3 neurons (left CA3 neuronal silencing, green) in learning of the reference memory Y-maze task is indistinguishable, but both are impaired relative to mice with delivery to right Arch3.0-eYFP-expressing CA3-CA1 afferents (right CA3-CA1 silencing, red).

Multi-way, repeated-measures ANOVA:

Main effect of group (left CA3 neuronal silencing, left CA3-CA1 silencing, right CA3-CA1 silencing): $F = 27.1$, $P < 0.001$.

Further analysis (t-test) showed no significant difference between left CA3 neuronal silencing and left CA3-CA1 silencing: $P = 0.431$, but a significant difference between left CA3 neuronal silencing and right CA3-CA1 silencing: $P < 0.001$.
